# Optimized Lung Nodule Classification Using CLAHE-Enhanced CT Imaging and Swin Transformer-Based Deep Feature Extraction

**DOI:** 10.3390/jimaging11100346

**Published:** 2025-10-04

**Authors:** Dorsaf Hrizi, Khaoula Tbarki, Sadok Elasmi

**Affiliations:** 1 COSIM Laboratory, Higher School of Communication of Tunis, University of Carthage, Ariana 2083, Tunisia; elasmi@supcom.tn; 2 National Institute of Technology and Science of Kef, University of Jendouba, El Kef 7100, Tunisia; khaoula.tbarki@gmail.com; 3 LR-SITI, National Engineering School of Tunis (ENIT), University of Tunis El Manar, Tunis 2092, Tunisia

**Keywords:** lung cancer, deep learning, swin transformer, image preprocessing, contrast limited adaptive histogram equalization, transfer learning, eXtreme Gradient Boosting

## Abstract

Lung cancer remains one of the most lethal cancers globally. Its early detection is vital to improving survival rates. In this work, we propose a hybrid computer-aided diagnosis (CAD) pipeline for lung cancer classification using Computed Tomography (CT) scan images. The proposed CAD pipeline integrates ten image preprocessing techniques and ten pretrained deep learning models for feature extraction including convolutional neural networks and transformer-based architectures, and four classical machine learning classifiers. Unlike traditional end-to-end deep learning systems, our approach decouples feature extraction from classification, enhancing interpretability and reducing the risk of overfitting. A total of 400 model configurations were evaluated to identify the optimal combination. The proposed approach was evaluated on the publicly available Lung Image Database Consortium and Image Database Resource Initiative dataset, which comprises 1018 thoracic CT scans annotated by four thoracic radiologists. For the classification task, the dataset included a total of 6568 images labeled as malignant and 4849 images labeled as benign. Experimental results show that the best performing pipeline, combining Contrast Limited Adaptive Histogram Equalization, Swin Transformer feature extraction, and eXtreme Gradient Boosting, achieved an accuracy of 95.8%.

## 1. Introduction

Lung cancer is one of the most aggressive and deadly cancers, responsible for 2.5 million new cases and 1.8 million deaths annually, the leading cause of cancer mortality worldwide [1,2]. Its poor prognosis results from the late diagnosis due to rapid progression, early metastases, and nonspecific symptoms. Early detection is essential; it greatly enhances prognosis.

Computed tomography (CT) is the primary tool for detecting pulmonary nodules. However, a radiologist’s interpretation is constrained by fatigue, varying expertise, and image complexity [3]. Computer-aided diagnosis (CAD) systems address these challenges by providing objective and rapid assessments. Recent advances in deep learning, particularly convolutional neural networks (CNNs) and transfer learning, have improved the performance of CAD by enabling automated feature extraction and leveraging pre-trained models on large datasets.

Nevertheless, the high variability of nodules, their similarity to benign structures, and the integration of segmentation into classification remain major obstacles. To overcome these challenges, this study presents a hybrid CAD system combining feature extraction through transfer learning and machine learning (ML) classifiers. This system is preceded by a preprocessing step, which improves image quality, reduces noise, and standardizes input data, thereby improving classification accuracy and efficiency. The proposed system aims to facilitate reliable early diagnosis of lung cancer in clinical settings.

The remainder of this paper is structured as follows. Section 2 reviews recent literature on lung cancer classification; Section 3 details the proposed methodology; Section 4 and Section 5 present the experimental results and discussion; and Section 6 concludes the study.

## 2. Related Works

Recent advances in deep learning (DL) and machine learning have significantly improved medical image analysis, particularly for lung cancer detection and classification. Various approaches combine deep architectures, transfer learning, and optimization strategies to enhance diagnostic accuracy.

Dechao Chen et al. [4] applied an optimized CNN using the Beetle Antenna Search algorithm for cerebral hemorrhage diagnosis, illustrating the benefit of optimization techniques. Vijayan et al. [5] evaluated six DL models such as AlexNet, GoogleNet, ResNet, Inception V3, EfficientNet B0, and SqueezeNet, finding that GoogleNet with the Adam optimizer achieved the highest performance, with 92.08% accuracy.

Kumar et al. [6] developed PneumoNet, an ensemble model for pneumothorax detection, reaching 98.41% accuracy. Nawreen et al. [7] highlighted hybrid pipelines combining edge detection, thresholding, and SVMs for tumor assessment. Ayad, Al-Jamimi, and El Kheir [8] proposed a hybrid RFE-SVM + XGBoost model, achieving 100% accuracy on two datasets, while Wang et al. [9] used residual networks with transfer learning for lung cancer subtypes, reaching 85.71% accuracy. Other notable studies include Sari et al. [10] (modified ResNet-50, 93.33% accuracy), Bakchy et al. [11] (lightweight CNN with Grad-CAM, 99.48% accuracy), Raza et al. [12] (LungEffNet using EfficientNet variants), and Fan and Bu [13] (DenseNet-121, 93.7% accuracy).

Hrizi et al. [14,15,16] developed a series of optimized CAD pipelines, culminating in a lightweight model achieving 97.06% accuracy in under 0.25 s. Idrees et al. [17] used a marker-controlled watershed algorithm for ROI identification, achieving 88.5% accuracy.

Xu et al. [18] proposed DCSAU-Net, a compact split-attention U-Net that preserves multiscale semantic features, outperforming state-of-the-art methods on CVC-ClinicDB, ISIC-2018, and BraTS-2021 in Dice score and mean IoU.

Liu et al. [19] developed a lightweight 3D CNN with attention mechanisms to classify lung nodule malignancy from CT scans, incorporating nodules and their fibrotic microenvironment, achieving 80.84% accuracy and an AUC of 0.89. Dhiaa and Awad [20] integrated transcriptomic data with XGBoost to predict postsurgical lung cancer recurrence, demonstrating the potential of multi-omics approaches. Li et al. [21] introduced a Swin Transformer–based dual-channel model combining CT and histopathological data to predict bone metastasis, achieving an AUC of 0.966 and outperforming ResNet50 baselines.

Despite these advances, challenges remain in generalization, model complexity, and clinical applicability. Many systems are dataset-specific and lack efficient integration of segmentation and classification. Few approaches combine transfer learning with lightweight classifiers for real-time use. To address these gaps, our study introduces a hybrid CAD system that unifies transfer learning–based feature extraction with classical ML classifiers, aiming for accurate and computationally efficient clinical diagnosis.

## 3. Lung Cancer Detection System

This work proposes a modular system for lung cancer classification, aimed at distinguishing benign from malignant pulmonary cases using chest CT images. The pipeline comprises three principal stages: (1) data preparation, (2) deep feature extraction, and (3) classification via machine learning models. Throughout these stages, various techniques were systematically evaluated to identify the optimal configuration for accurate classification. Overall, four hundred distinct combinations were examined.

**Figure 1:**
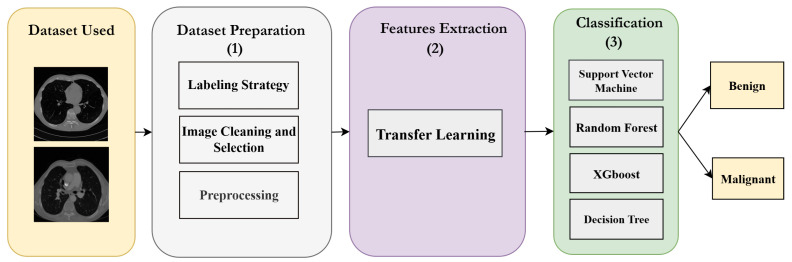
Pipeline architecture of the proposed lung cancer detection system.

The overall architecture of our proposed methodology is illustrated in Figure 1.

### 3.1. Dataset

The publicly available Lung Image Database Consortium and Image Database Resource Initiative (LIDC-IDRI) dataset from The Cancer Imaging Archive was used in this study. This collection contains 1018 thoracic CT scans, each annotated by four thoracic radiologists through a rigorous two-phase process. The first phase was conducted in a blinded manner to identify lung nodules, while the second phase involved an unblinded review to refine the assessments, deliberately preserving inter-observer variability.

### 3.2. Dataset Preparation

The dataset preparation process comprised two main steps: Section 3.2.1 The labeling of images step based on radiologist’s scores and Section 3.2.2 the preprocessing step to enhance image quality for subsequent analysis.

#### 3.2.1. Labeling Strategy

The labeling strategy was based on the malignancy scores provided by radiologists in the LIDC-IDRI dataset. Nodules with an average score of 2 or lower were classified as benign, whereas those with an average score of 4 or higher were classified as malignant. Nodules with scores around 3 were excluded to avoid ambiguity. At the patient (scan) level, the following rules were applied. If all nodules in a scan were benign, the case was labeled benign, and if at least one nodule was malignant, the entire scan was labeled malignant.

This labeling strategy ensured consistency between nodule-level and patient-level annotations, while also reflecting clinical practice, where the presence of a single malignant lesion determines the overall diagnosis.

#### 3.2.2. Preprocessing

To improve the quality and diagnostic value of the medical images prior to feature extraction, a comprehensive set of ten preprocessing techniques were applied. These techniques, selected based on literature prevalence and clinical relevance as well as complementary technical roles, aim to enhance contrast, suppress noise, correct imaging artifacts, and emphasize relevant anatomical structures. In this section, we categorize the preprocessing techniques into three functional groups: contrast enhancement, noise reduction, and structure highlighting.

(a) Histogram-Based Enhancement
  Two prominent techniques within this category are Histogram Equalization (HE) and Contrast Limited Adaptive Histogram Equalization (CLAHE); both of them have shown significant potential in improving the quality and diagnostic value of medical images.
  **HE:** Redistributes pixel intensity values across the histogram to enhance global contrast, improving the visibility of overall features, such as in the work of Sunanda and Rani [22].
  **CLAHE**: Unlike standard HE, CLAHE operates on small image tiles and limits contrast amplification to avoid noise, highlighting local structures such as tissue boundaries [23].
(b) Intensity and Illumination Correction
  Techniques under this category aim to correct uneven illumination and intensity artifacts, which are common in medical imaging and can hinder accurate analysis.
  **Logarithmic Transformation**: Enhances low-intensity pixels, emphasizing faint structures and low-contrast details. This transformation was also employed in the work of Domain-Aware Adaptive Logarithmic Transformation [24].
  **Intensity Inhomogeneity Correction**: Compensates for spatial intensity variations (bias fields) in MRI, improving contrast uniformity and supporting tissue segmentation or lesion detection [25].
(c) Edge and Structure Enhancement
  Edge and structure enhancement techniques aim to highlight important anatomical contours and assist in the preliminary localization of regions of interest, which are crucial for subsequent diagnostic tasks.
  **Edge and Contour Enhancement**: Filters and algorithms highlight anatomical boundaries without increasing noise [26].
  **Preliminary Segmentation**: Thresholding separates regions of interest from the background to guide further analysis [27]
(d) Noise Reduction and Filtering
  Effective noise suppression is a crucial preprocessing step in medical image analysis, and several filtering techniques including linear, non-linear, and multiscale methods are commonly used to address this challenge.
  **Gaussian Filter**: Suppresses high-frequency noise while preserving structure [28].
  **Median Filter**: Removes salt-and-pepper noise without blurring edges [29].
  **Anisotropic Filtering**: Smooths images while preserving edges through gradient-based diffusion [30].
  **Wavelet Based Denoising**: Multi-resolution transforms separate noise from signal, allowing scale-specific noise suppression while maintaining edges [31].

Each technique was evaluated independently to assess its impact on image clarity, contrast, and downstream model performance, guiding the selection of the most effective preprocessing combination.

### 3.3. Feature Extraction

Following the preprocessing step, features were extracted using ten Transfer learning architectures: Residual Network (ResNet50), InceptionV3, Densely Connected Convolutional Network (DenseNet121), EfficientNet (B0, B1, B3), Swin Transformer, Denoising Autoencoder, Variational Autoencoder, and Convolutional Autoencoder (CAE). Input images were resized to match model requirements, and feature vectors were extracted from the penultimate layer for subsequent machine learning classification. This setup allowed evaluation of how different preprocessing and feature extraction combinations affect classification of benign and malignant cases. **ResNet50** uses residual connections, **InceptionV3** captures multi-scale features, **DenseNet121** strengthens inter-layer information flow, and **EfficientNet** variants balance depth, width, and resolution. Denoising and Variational **Autoencoders** enhance robustness and generalization, while **CAE** captures spatial features in a compact latent representation.

Although ten deep learning models were explored for feature extraction, we describe in detail the architectures of the Swin Transformer and the CAE, as they combine strong performance with distinctive design principles and were among the most successful models in our experiments. The Swin Transformer serves as a representative example for the feature extraction process in this study, while the CAE demonstrates strengths in unsupervised spatial feature learning. The other architectures used in this study are well established and extensively described in the literature. For instance, ResNet50 [32], InceptionV3 [33], DenseNet121 [34], and EfficientNet (B0–B2) [35] are widely adopted convolutional neural networks, especially in transfer learning contexts. The Denoising Autoencoder and Variational Autoencoder are also standard architectures in unsupervised learning and have been applied successfully in medical imaging [36]. The CAE combines spatial feature extraction with compact latent encoding through a bottleneck structure [37]. Readers are referred to relevant studies, including [38], which illustrate similar use of transfer learning and CNN-based models in medical diagnosis tasks.

(a) **Swin Transformer**

To ensure compatibility with the Swin Transformer model pre-trained on ImageNet, grayscale images were converted to three-channel RGB images by duplicating the single channel.

**Figure 2:**
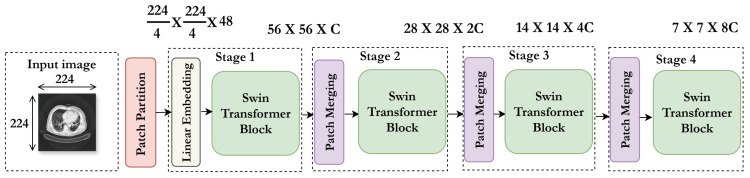
Swin Transformer architecture for feature extraction.

The architecture, illustrated in Figure 2, processes images in four stages. In the first stage, the input RGB image (224 × 224) is split into non-overlapping **4 × 4 patches**, resulting in **56 × 56 patches** with **48 channels** each. Next, patches are organized into windows where self-attention is applied locally to capture spatial dependencies. The third stage shifts window positions to create overlaps, enabling interactions between adjacent windows. Finally, features are progressively merged in a hierarchical aggregation to combine local details with global context.

This hierarchical structure maintains computational efficiency while progressively refining feature extraction.

(b) **Convolutional Autoencoder**

The CAE is specifically designed for high-resolution grayscale CT images (512 × 512 pixels) and operates in an unsupervised learning system. It consists of two main parts:

**The encoder** captures hierarchical spatial features and compresses them into a compact latent space. Its architecture consists of three convolutional blocks. Each block uses increasing filter sizes, starting with 32, then 64, and finally 128. Following each convolutional block, a **2 × 2 max-pooling layer** with same padding is applied. This progressive reduction in spatial resolution helps to preserve important anatomical details and results in a bottleneck feature map of **64 × 64 × 128**. This final feature map effectively encodes both local texture and global structural information.

**The decoder** is responsible for reconstructing the input from the compact latent representation generated by the encoder. Its architecture mirrors the encoder’s, using upsampling and convolutional layers to reverse the encoding process and restore the original image.

**Figure 3:**
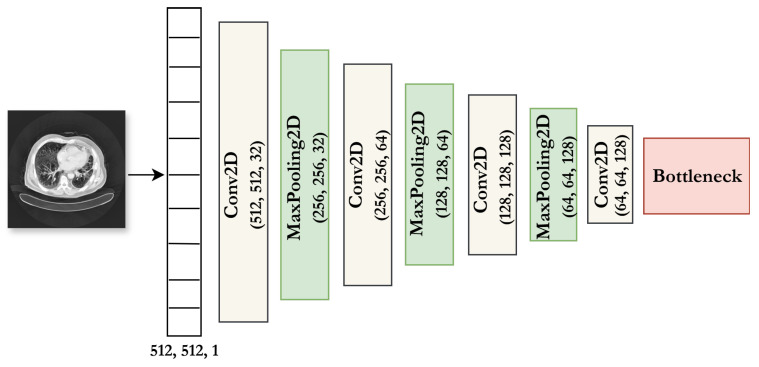
Architecture of the Convolutional Autoencoder (CAE) used for feature extraction from 512 × 512 CT images.

For feature extraction, only the encoder is used. The 64 × 64 × 128 bottleneck is flattened to form a vector of 524,288 features, which is then stored in CSV format and passed to classical classifiers. The architecture of the CAE used in this study is illustrated in Figure 3.

### 3.4. Classification

The feature vectors extracted from the deep learning models were classified using four supervised machine learning algorithms, selected for their proven efficiency in handling high-dimensional data and their frequent use in medical image classification.

**Support Vector Machine (SVM)** is well suited for datasets with high-dimensional feature spaces, as it identifies the optimal hyperplane maximizing class separation. Its robustness and generalization make it reliable for medical decision making.

**Random Forest (RF)** builds multiple decision trees on random subsets of data and features, aggregating outputs via majority voting. This ensemble approach reduces overfitting and handles noisy or redundant features effectively.

**Decision Tree (DT)** is simpler and more interpretable than ensemble methods, providing rapid inference. Although prone to overfitting with high-dimensional data, it serves as a useful baseline.

**eXtreme Gradient Boosting (XGBoost)** sequentially constructs trees, optimizing performance by correcting previous errors. Its regularization and efficiency make it a strong alternative to RF and SVM.

Each classifier was tasked with distinguishing between two lung cancer categories: benign and malignant. To ensure fairness and reproducibility, stratified train–test splits were employed to preserve class distributions.

Performance was primarily assessed using classification accuracy, which reflects the proportion of correctly predicted samples. Although additional metrics such as precision and recall provide further clinical insights, accuracy was chosen here to enable consistent comparison across all preprocessing, feature extraction, and classification configurations.

## 4. Results

This section presents the outcomes of the experimental pipeline described previously. The performance of each classification model was evaluated across the various combinations of preprocessing techniques and feature extraction methods. The results are organized to highlight the influence of (1) data preparation, (2) feature extraction models, and (3) classification algorithms on the accuracy of medical image classification into benign or malignant cases.

### 4.1. Dataset Preparation Results

The following two subsections present the results of labeling and preprocessing.

#### 4.1.1. Labeling Strategy Results

After applying the labeling strategy described in Section 3.2.1, the initial set of annotated images from the LIDC-IDRI dataset was transformed into a consistent binary classification problem. The distribution of images across the different categories is summarized in Table 1.

#### 4.1.2. Preprocessing Results

Among the various preprocessing techniques evaluated in our study, CLAHE demonstrated the most effective results. CLAHE significantly improved the visibility of structural details within the CT images by enhancing local contrast, particularly in regions with low intensity variation. One of the key advantages of CLAHE is its ability to normalize image intensities across different scans, which helps to mitigate the impact of illumination inconsistencies and intensity inhomogeneities.

This normalization effect is particularly beneficial in the context of transfer learning, as it ensures that the input features are more consistent and representative, thereby facilitating more robust and accurate feature extraction by deep learning models. Consequently, CLAHE was selected as the optimal preprocessing method for our classification pipeline. The innovative aspect of this study lies in the exhaustive and systematic evaluation of the combined effect of eleven preprocessing techniques on image quality and the performance of feature extraction and classification models. This comprehensive approach allows precise identification of the most effective combinations, which is rarely explored in current literature.

Furthermore, we visually compared the outcomes of all preprocessing techniques on three representative samples from the dataset in Table 2. This visual analysis helped to qualitatively validate the effectiveness of CLAHE in enhancing image quality across different pathological conditions.

To quantitatively assess the contribution of each preprocessing method to classification performance, we computed the average accuracy achieved across all combinations of feature extractors and classifiers for each technique. Table 3 summarizes these average accuracies. This analysis supports the visual findings and further highlights the superiority of CLAHE in consistently enhancing downstream classification results.

### 4.2. Feature Extraction Results

In our study, we evaluated several deep learning models for feature extraction, including VGG16, ResNet50, InceptionV3, and the Swin Transformer. Among these, the Swin Transformer was selected for detailed analysis due to its strong ability to model both local and global image dependencies.

Unlike traditional convolutional neural networks that operate with fixed receptive fields, the Swin Transformer uses a hierarchical architecture with shifted windows, allowing it to efficiently capture fine-grained local structures as well as broader contextual information. This is particularly advantageous in medical imaging tasks such as lung cancer detection, where subtle texture patterns and spatial relationships between anatomical regions are critical. The Swin Transformer’s capacity to maintain high-resolution feature representations while progressively aggregating context makes it especially powerful for extracting rich and discriminative features from CT images.

**Figure 4:**
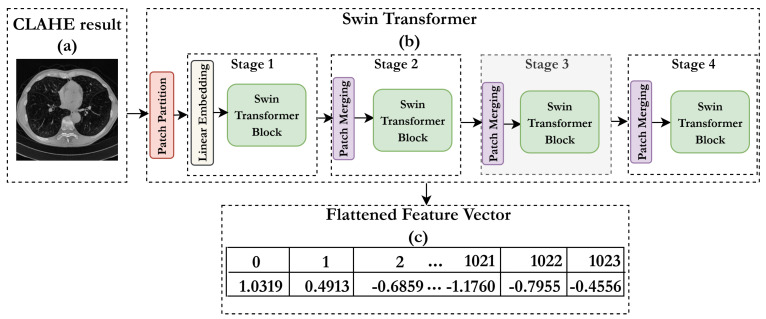
Example of feature extraction process. (**a**) CLAHE-enhanced CT slice. (**b**) Simplified representation of the Swin Transformer feature extraction pipeline, showing consecutive Swin Transformer blocks across four stages. (**c**) Flattened feature vector (size: 1 × 1024) obtained from the final stage; only the first five and last four feature values are displayed for illustration.

After applying the CLAHE preprocessing technique, each CT scan image was resized to a standard dimension of 512 × 512 pixels to ensure uniformity across the dataset. When processed through the Swin Transformer model, each preprocessed image yielded a feature vector of 1024 dimensions, capturing both local and global characteristics of the lung region. In total, we obtained 11,417 labeled samples corresponding to the two classes: malignant and benign. These extracted features, illustrated in Figure 4, served as the input for the subsequent classification stage.

### 4.3. Classification Results

In the binary classification task distinguishing benign from malignant lung nodules, the XGBoost classifier demonstrated the strongest performance. Its gradient boosting framework builds decision trees sequentially to minimize classification error, effectively capturing complex feature interactions while controlling overfitting through regularization. This makes XGBoost particularly well suited for structured features extracted from deep learning models.

Random Forest ranked second, benefiting from its ensemble of decision trees that reduce variance and improve generalization. However, its averaging nature can smooth critical decision boundaries, which may slightly limit performance in subtle distinctions between benign and malignant nodules.

Decision Tree showed moderate performance due to its limited ability to model complex patterns, while SVM, despite its theoretical strength in high-dimensional spaces, achieved the lowest accuracy in this binary setting.

Table 4 shows that the XGBoost classifier achieves outstanding class-specific performance in differentiating benign and malignant lung nodules. For the Benign class, the model achieved a precision of 91.0%, a recall of 93.0%, and an F1-score of 92.0%, indicating that most nodules predicted as benign were correct and that the majority of benign nodules were successfully identified. For the Malignant class, the classifier showed even higher performance, with a precision of 97.0%, a recall of 98.0%, and an F1-score of 97.5%, reflecting strong reliability in detecting malignant tumors. Overall, these results highlight XGBoost’s robustness in handling class imbalance and maintaining high diagnostic accuracy, making it highly effective for binary classification of lung nodules.

(a) **Statistical Significance Analysis**

To determine whether the observed differences in F1-scores between the evaluated configurations were statistically significant, we performed a two step non-parametric analysis using the five-fold cross-validation results reported in Table 5 for all CLAHE-based configurations.

First, a Friedman test was applied to the complete set of configurations (all combinations of feature extraction models and classifiers using CLAHE preprocessing). The null hypothesis stated that all models had equal median performance. The Friedman test revealed a statistically significant difference among the configurations (p<0.001), indicating that at least one configuration outperformed the others.

Second, post hoc pairwise comparisons were conducted using the Wilcoxon signed-rank test with Bonferroni correction to control for multiple comparisons. In this step, the best-performing configuration (CLAHE + Swin Transformer + XGBoost) was compared individually to each of the other configurations. The results are presented in Table 6. The proposed CLAHE + Swin Transformer + XGBoost pipeline achieved significantly higher F1-scores than all other configurations (p<0.01).

These results provide strong statistical evidence that the proposed configuration not only achieves the highest average F1-score but also delivers a performance advantage that is statistically significant compared to most alternative pipelines tested in this study.

## 5. Discussion

The experimental results of our study demonstrate that the proposed hybrid pipeline achieved a classification accuracy of 95.8% on the LIDCI-IDRI dataset, confirming its competitiveness with current state-of-the-art methods in lung cancer detection. Notably, the ExtRanFS framework [39] and the attention-enhanced InceptionNeXt model [40] used the IQ-OTH/NCCD dataset and reported accuracies of 99.09% and 99.54%, respectively. While these approaches rely on tightly coupled end-to-end architectures combining CNNs, Vision Transformers, and tree-based or deep classifiers, our method introduces a modular design that decouples preprocessing, feature extraction, and classification. This strategy provides greater interpretability, robustness, and the flexibility to explore a wide range of configurations for optimized performance.

Unlike these methods, which focus on specific architectures or fused attention mechanisms, our pipeline evaluates 400 distinct combinations, integrating 10 preprocessing techniques, 10 pretrained deep models (including CNNs and transformer-based architectures), and 4 classical machine learning classifiers. This comprehensive and systematic exploration enabled the identification of the best-performing configuration (CLAHE + Swin Transformer + XGBoost), while also offering valuable insights into the contribution of each component. The use of Swin Transformer for feature extraction brings the advantage of modeling both local and global dependencies in CT images, and its combination with a lightweight, explainable classifier like XGBoost reduces overfitting risks.

In contrast, the study presented in [41] proposes a hybrid algorithm that combines SURF feature extraction, genetic optimization, and a Feed-Forward Back Propagation Neural Network (FFBPNN), achieving an accuracy of 98.08%. While promising, this approach is based on handcrafted feature engineering and limited data volume (500 images), which may limit its generalizability compared to deep feature-based models.

As for [42], although it focuses on a different lung condition (pneumothorax) and a different imaging modality (chest X-rays), its lower performance (91.23% accuracy, 92.20% F1-score) further illustrates the challenges of using 2D X-ray data for accurate diagnosis of thoracic pathologies and highlights the relevance of CT imaging in this context.

In summary, our method offers a strong balance between accuracy, modularity, and interpretability. It is particularly well suited for adaptation to clinical settings where understanding the contribution of each module (preprocessing, feature extraction, classification) is critical. Moreover, the flexibility of our pipeline allows easy integration of future models or preprocessing enhancements.

Nevertheless, the study has certain limitations. It was conducted on a single dataset, and further evaluation on external datasets is needed to validate generalizability. Clinical validation and integration with patient metadata are also part of our future work.

## 6. Conclusions

Unlike traditional deep learning approaches that rely solely on pre-trained models for both feature extraction and classification, our proposed system adopts a hybrid architecture that separates these two stages. Specifically, we leverage pre-trained models to extract deep, high-level feature representations from CT scan images, and subsequently feed these features into classical machine learning classifiers such as SVM, XGBoost, and Random Forest.

This design offers several key advantages. First, it allows for better control and interpretability of the classification stage, enabling fine-tuned optimization of decision boundaries through well-established machine learning algorithms. Second, it reduces the risk of overfitting, by avoiding the need to retrain the final fully connected layers of deep models.

Furthermore, our system enables modular experimentation with different preprocessing techniques and classifiers, allowing the construction of a flexible and extensible diagnostic system. Remarkably, the combination of CLAHE preprocessing, Swin Transformer feature extraction, and XGBoost with a classification strategy achieved the highest accuracy of 95.8%, highlighting the effectiveness of our lung cancer detection system in distinguishing between malignant and benign lung CT scans with high precision.

## Figures and Tables

**Table 1:** Distribution of images after applying the labeling strategy.

Class	Number of Images
Malignant	6568
Benign	4849

**Table 2:** Visual comparison of different preprocessing techniques.

Preprocessing Technique	Benign Case	Malignant Case
Original image	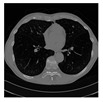	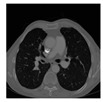
Edge and contour enhancement	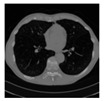	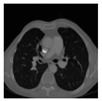
Histogram equalization	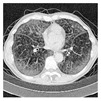	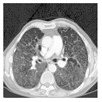
CLAHE	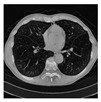	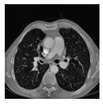
Intensity inhomogeneity correction	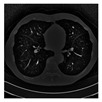	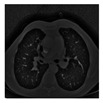
Median filter	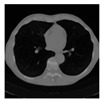	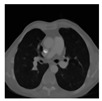
Gaussian filter	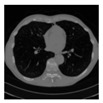	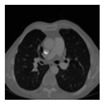
Logarithmic transformation	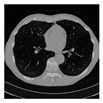	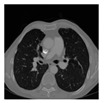
Anisotropic filter	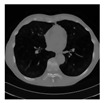	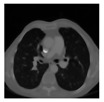
Preliminary segmentation	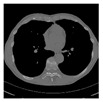	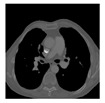
Wavelet-based denoising	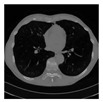	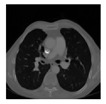

**Table 3:** Average classification accuracy per preprocessing method across all configurations.

Preprocessing Technique	Average Accuracy (%)
Original Image	84.3
Histogram Equalization	78.2
CLAHE	**89.0**
Intensity Inhomogeneity Correction	78.4
Median Filter	76.6
Gaussian Filter	80.5
Logarithmic Transformation	79.3
Anisotropic Filter	78.8
Preliminary Segmentation	79.1
Wavelet-Based Denoising	76.6
Edge and contour enhancment	80.1

**Table 4:** Class-wise performance metrics of the XGBoost classifier.

Class	Precision (%)	Recall (%)	F1-Score (%)	Support
Benign	91.0	93.0	92.0	1455
Malignant	97.0	98.0	97.5	1971

**Table 5:** Combinations of preprocessing techniques with feature extraction models and classification results.

Preprocessing	Feature Extraction Model	SVM			RF			DT			XGBoost		
		Acc.	Brier Score Loss	F1	Acc.	Brier Score Loss	F1	Acc.	Brier Score Loss	F1	Acc.	Brier Score Loss	F1
CLAHE	ResNet50	85.88	0.18	0.80	86.82	0.16	0.84	74.34	0.25	0.77	87.39	0.15	0.85
	InceptionV3	86.94	0.14	0.87	79.45	0.22	0.86	85.63	0.18	0.80	82.53	0.20	0.88
	DenseNet121	89.22	0.12	0.86	83.18	0.19	0.83	74.57	0.27	0.71	81.96	0.21	0.82
	EfficientNet B0	87.73	0.13	0.86	85.63	0.16	0.85	79.48	0.23	0.70	85.24	0.15	0.85
	EfficientNet B1	88.65	0.11	0.84	87.69	0.14	0.87	70.53	0.28	0.70	86.73	0.13	0.87
	EfficientNet B3	87.77	0.12	0.87	84.6	0.18	0.84	65.89	0.30	0.66	83.88	0.19	0.84
	**Swin Transformer**	90.0	0.08	0.89	91	0.07	0.90	92.0	0.06	0.91	**95.8**	0.04	0.94
	Denoising Autoencoder	78.5	0.23	0.76	85.0	0.17	0.80	91.8	0.09	0.90	87.0	0.15	0.85
	Variational Autoencoder	83.66	0.19	0.81	80	0.22	0.79	79.26	0.24	0.78	78.23	0.25	0.77
	Convolutional Autoencoder	71.26	0.29	0.71	91.45	0.08	0.91	82.29	0.20	0.82	91.23	0.09	0.91

**Table 6:** Post hoc pairwise Wilcoxon signed-rank test with Bonferroni correction for CLAHE preprocessing (*p*-values).

Model A	Model B	*p*-Value
Swin Transformer + XGBoost	ResNet50 + XGBoost	0.001
Swin Transformer + XGBoost	DenseNet121 + XGBoost	0.0005
Swin Transformer + XGBoost	EfficientNet B3 + XGBoost	0.0002
Swin Transformer + XGBoost	Denoising Autoencoder + XGBoost	0.0001
Swin Transformer + XGBoost	Variational Autoencoder + XGBoost	0.0001
Swin Transformer + XGBoost	Convolutional Autoencoder + XGBoost	0.045

## Data Availability

The original data presented in the study are openly available in LIDC-IDRI dataset at https://www.cancerimagingarchive.net/collection/lidc-idri/ (accessed on 29 September 2025).
